# Endodontic Management of a C‐Shaped Maxillary Second Molar With Five Canals: A Case Report

**DOI:** 10.1155/crid/8867290

**Published:** 2026-06-14

**Authors:** Yiwen Wang, Beining Liao, Hongyin Ruan, Xinyan Ge, Dan Zhao

**Affiliations:** ^1^ State Key Laboratory of Oral & Maxillofacial Reconstruction and Regeneration, Key Laboratory of Oral Biomedicine Ministry of Education, Hubei Key Laboratory of Stomatology, School & Hospital of Stomatology, Wuhan University, Wuhan, China, whu.edu.cn; ^2^ Department of Oral Health Sciences, Population Studies in Oral Health, KU Leuven, Leuven, Belgium, kuleuven.be

**Keywords:** cone-beam computed tomography, C-shaped, maxillary molar, root canal treatment

## Abstract

The understanding of morphological variations in the canal along with the ability to detect and manage the root canal system are essential for achieving a satisfactory endodontic treatment outcome. This case report presents the endodontic management of a C‐shaped maxillary second molar with five canals. Cone‐beam computerised tomography coupled with a dental operating microscope exhibited remarkable efficacy in the diagnosis, detection and treatment of this irregular morphological root canal system. Clinicians should be alert to anatomic variations in C‐shaped maxillary molars during endodontic treatment, even though these variations rarely occur.

## 1. Introduction

A comprehensive awareness of root canal morphological diversity is crucial for favourable endodontic therapy [1, 2]. The term ‘C‐shaped canal’ was firstly introduced in 1979 to describe a canal resembling the capital letter ‘C’ [3]. The core morphological characteristic of C‐shaped canals lies in the existence of a fin or web between canals. A root canal′s orifice is a single band‐like structure with an arc angle of ≥ 180°, influencing the cross‐sectional shape and three‐dimensional (3D) morphology of the canal [4]. When recognised, the C‐shaped canal complicates debridement and obturation because of its morphological complexity [5].

A C‐shaped canal is predominantly found in the mandibular second molar [1]. However, this configuration has also been observed in maxillary molars [6, 7]. Mashyakhy et al.′s [8] study on the population of Saudi Arabia revealed 1.1% prevalence rate of C‐shaped canals in the maxillary second molars (MSMs) and the greater morphological complexity in the MSMs compared with maxillary first molars. Martins et al. [9] found that the incidence rate of C‐shaped canals within MSMs reached 3.8%; they also expressed interest in a similar epidemiological study in Asian populations, which showed a high frequency of this anatomical variation. The C‐shaped canals of the mandibular second molars are highly prevalent (39%–47.96%) in a Chinese population [1, 4]; however, the C‐shaped canals of the MSMs in this group are rarely studied [10]. The limited data were generated by Yang et al. [11] and Qian et al. [12]. Yang et al. [11] employed a clearing technique and identified a 4.9% C‐shaped canal prevalence in Chinese MSMs, and Qian et al. [12] found C‐shaped root canals in 5.24% of MSMs in the Southwestern Chinese population. The low incidence (1.1%–5.24%) in these studies [8, 9, 11, 12] suggests that the C‐shaped root canal anatomy of MSMs can be irregular and unpredictable in clinical practise.

A full preoperative understanding of anatomical root canal features can help with the efficient endodontic treatment of the variable canal systems in MSMs. Proper radiographic examination is critical to recognise C‐shaped canals and achieve successful endodontic management. Cone‐beam computerised tomography (CBCT) imaging provides accurate 3D anatomical details to detect C‐shaped canals in different regions [13–15]. CBCT imaging has lower radiation doses and is more feasible for clinical application compared with traditional computed tomography imaging [16]. Limited‐field‐of‐view CBCT was recommended as the imaging modality for the initial endodontic treatment of teeth with suspected complex canal morphology [17, 18].

This case report presents the endodontic management of a C‐shaped MSM, diagnosed and confirmed using CBCT. The 3D reconstruction of CBCT images enables the visualisation of the root canal variations of MSMs and favourable endodontic management.

## 2. Case Report

**Figure 1:**
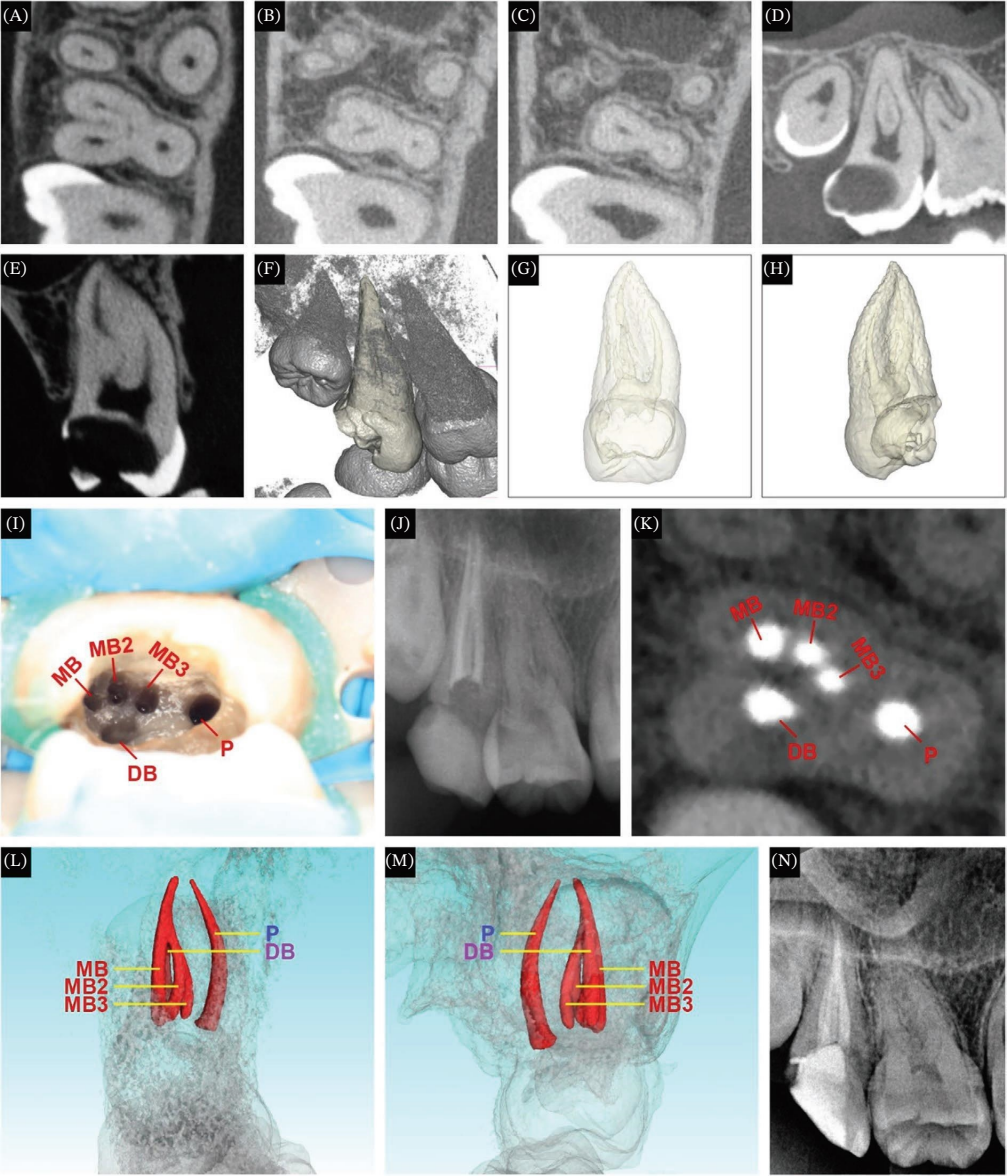
Endodontic treatment of a C‐shaped MSM with five canals: (A) preoperative CBCT scanning image in axial view at coronal third root; (B) preoperative CBCT image in axial view at middle third root; (C) preoperative CBCT image in axial view at apical third root; (D) preoperative CBCT image in coronal view; (E) preoperative CBCT image in sagittal view. (F–H) 3D reconstructions of preoperative CBCT images indicating root fusion; (I) five root canal orifices of MB, MB2, MB3, DB and P; (J) postoperative radiograph of root canal obturation; (K) 4‐month follow‐up CBCT image indicating the five root canal orifices of MB, MB2, MB3, DB and P; (L) 3D reconstruction of the 4‐month follow‐up CBCT image illustrating MB2 and MB3 fused at the coronal third canal; (M) 3D reconstruction of the 4‐month follow‐up CBCT image illustrating that MB, MB2, MB3 and DB fused at the apical third of the canal and ultimately went into a unified apical foramen; (N) 6‐year follow‐up radiograph showed no periapical radiolucency of the right maxillary second molar.

A 27‐year‐old female visited the Department of Dental Emergency in the Stomatological Hospital of Wuhan University (Wuhan, China) for spontaneous pain at her right facial area for 4 days. Her medical history was unremarkable. Clinical examination demonstrated a deep carious lesion on her right MSM. The molar was tender to percussion and exhibited significant sensitivity to thermal stimuli and electrical pulp test. The molar was diagnosed with symptomatic irreversible pulpitis and scheduled for endodontic therapy. A preoperative radiographic examination using limited‐field‐of‐view CBCT (3D Accuitomo, J. Morita MFG. Corp, Kyoto, Japan) was performed using the following parameters: 70 kV, 3 mA, 8 cm∗8 cm field of view, 0.16 mm^3^ voxel size and 0.16 mm layer thickness. Radiographic examination demonstrated the mesiobuccal (MB) canal and distobuccal (DB) canal fused to form a semilunar root canal in this right MSM (Figures 1A–C). Following the criterion defined by Martins et al. [9], this case was classified as type B of maxillary C‐shaped molars showing the fusion of MB and DB canals. The 3D reconstructions of preoperative CBCT images revealed that all roots fused. (Figures 1F–H).

After receiving an explanation of the process and complications of root canal treatment, the patient signed the agreement to accept the treatment. Under isolation using a rubber dam, the tooth was locally anaesthetised with 4% articaine supplemented with epinephrine at a concentration of 1:100,000 (Septocaine, Septodont, Saint‐Maur‐des‐Fosse, France). Following the removal of caries and the establishment of endodontic access, the following five canal orifices were identified using a Hu‐Friedy DG 16 endodontic explorer (Hu‐Friedy, Chicago, Illinois, United States) and dental operating microscope (ProErgo, Zeiss, Oberkochen, Germany) (Figure 1I): MB, MB two (MB2), MB three (MB3), DB and palatal (P). Except for MB2 and MB3 which exhibited slight calcification, all canals were initially suitable for establishing a full working length with a size 10 K‐file via an electronic apex locator (Propex II, Dentsply Maillefer, Ballaigues, Switzerland). The coronal part of the calcified root canals was enlarged with the ET 20 ultrasound tip (Satelec, Mérignac, France) under a dental microscope. The canals were finally negotiated successfully with small, prebent hand files (C‐Pilot Files Size 06 and 08; VDW) via the balanced force technique [19]. After the working length for all five root canals was reached, the canals were prepared with ProTaper Universal files (Dentsply Maillefer, Ballaigues, Switzerland) to Size #F1 in the MB2 and MB3 canals; Size #F2 in the MB and DB canals; and Size #F3 in the P canals [20]. During the instrumentation, 20 mL of 5.25% sodium hypochlorite (NaOCl) and 20 mL of 17% ethylenediaminetetraacetic acid (EDTA) were applied for canal irrigation. After root canal instrumentation, supplementary passive ultrasonic irrigation (Satelec Acteon, Mérignac, France) was performed three times for 20 s within each canal. Calcium hydroxide paste was used as an intracanal medicament for 1 week. One week later, the tooth exhibited no symptoms. The entire canal system was irrigated with 5.25% NaOCl and 17% EDTA, followed by passive ultrasonic irrigation and obturation using the warm vertical compaction technique with AH Plus (Dentsply De Trey GmbH, Konstanz, Germany) as the sealing cement and the Elements Obturation Unit (SybroEndo, Glendora, California) (Figure 1J). The tooth was filled with composite resin (Gradia Direct A3, GC Corporation, Tokyo, Japan) and recommended for full crown protection.

During the 4‐month follow‐up, the patient reported no clinical discomfort. CBCT radiographic examinations demonstrated no significant abnormalities in the affected tooth and its periapical area. The reconstruction of CBCT images revealed that MB2 and MB3 fused at the coronal third canal (Figure 1L). Subsequently, MB, MB2, MB3 and DB fused at the apical third of the canal and ultimately went into a unified apical foramen (Figure 1M).

At the 6‐year follow‐up, the patient reported no clinical discomfort, and the radiograph showed no periapical radiolucency of the affected tooth (Figure 1N).

## 3. Discussion

Prior knowledge of the anatomical features of root canals contributes to efficient debridement and disinfection during endodontic treatment [21, 22]. C‐shaped canals usually demonstrate high diversity and complexity, presenting considerable challenges to the successful outcome of endodontic treatment. Although C‐shaped canals are predominantly observed in mandibular molars [1, 4], their rare prevalence in maxillary molars has been documented [8, 9, 11, 12]. A limited number of published clinical cases [23–26] revealed the difficulty of nonsurgical endodontic treatment for C‐shaped maxillary molars. Additionally, studies mostly focused on the configuration of the P canal [23, 25, 26] and lacked 3D reconstruction images to depict the anatomical complexity of the root canal system [23, 24, 26]. This work presents a case of C‐shaped MSM with five canals and provides an intuitive demonstration of its C‐shaped morphology via 3D tooth reconstruction.

Qian et al. [12] indicated that the predominant type of C‐shaped maxillary molars in the Southwestern Chinese population is the fusion of MB and DB canals, corresponding to type B in the present case. For other populations, Martins et al. [9] also identified Type B as the most prevalent within the C‐shaped canal system of MSMs. By contrast, Mashyakhy et al. [8] reported no Type B in C‐shaped MSMs amongst a Saudi Arabian population. The difference might be due to regional population diversity.

When accompanied with C‐shaped canals, the diagnosis and endodontic management of the root canal system become highly challenging. Previous studies demonstrated that CBCT is helpful in identifying C‐shaped maxillary molars [24, 25]. In the current case, the C configuration observed in the cross‐sections of CBCT images, coupled with root fusion evident in the 3D reconstruction of preoperative CBCT images, attracted the attention of the clinician before the operation. When recognised, the C‐shaped morphology gives rise to a challenge in the root canal treatment of maxillary molars. Firstly, MSMs are located towards the end of the upper jaw, leading to operational stress. The existence of a ribbon‐shaped opening brings out difficulties in the detection of all root canal orifices. Carefully exploring the canal orifice with a Hu‐Friedy DG 16 endodontic explorer under a dental microscope, starting from the beginning of the ribbon‐shaped opening in a clockwise or anticlockwise direction, is suggested to avoid omissions [27]. Secondly, the extra canals usually demonstrate calcification and cause difficulty in reaching the full working length. In the present case, the ET 20 ultrasound tip was used to enlarge the coronal part of the calcified extra root canals under the dental microscope, and the calcified canals were finally negotiated successfully with small, prebent hand files via the balanced force technique. Thirdly, the irregular regions of the C‐shaped morphology contain soft‐tissue residuals or infected debris that may evade cleaning and filling during canal treatment. Therefore, extra efforts are required to achieve successful treatment [7]. Meanwhile, the excessive use of instruments that may lead to perforation should be avoided [22]. In the present case, ultrasonic irrigation was employed to efficiently clean the isthmuses, and the warm vertical compaction technique was used to seal the narrow isthmuses [22]. Vertical (warm) condensation allows the gutta‐percha to flow into the root canal system, achieving better adaptation in fins, isthmuses and irregularities compared with lateral condensation or compaction (mechanical) condensation [27, 28]. In this work, 3D reconstruction of 4‐month follow‐up CBCT revealed favourable 3D obturation results of isthmuses in the C‐shaped canal system.

Detecting all the canals in the root canal system is crucial for a case′s eventual success [29]. The presence of the MB root with three canals of the MSMs is relatively uncommon. In their vitro study, Sert and Bayirli [30] observed a 1.3% prevalence of this variation in the maxillary molars of a Turkish population. Ahmad and Al‐Jadaa [20] demonstrated a 1.3%–2.4% occurrence of three‐canal MB root in maxillary molars. Missing roots or root canals is the primary reason for unsuccessful root canal therapy [29]. A clinician should be alert to rare canal anatomies and avoid limiting themselves to conventional root canal configuration [29]. The combined application of CBCT imaging and a dental operating microscope has been proven to help locate extra canals [23]. Additionally, this case involved a 27‐year‐old female patient. The prevalence of supernumerary root canals in individuals younger than 40 years is greater than that in older populations because of the age‐related calcification of root canal orifice in elderly patients [20].

The present case report has certain limitations. Firstly, the relatively short follow‐up period limited the assessment of long‐term outcomes. Secondly, the limitation of CBCT resolution in this case prevented complete presentation of the entire canal system. Thirdly, because of the limitation of limited‐field‐of‐view CBCT in the study, only the right maxillary second molar and its adjacent teeth were visualised in the CBCT, which restricted the evaluation of the bilateral symmetry of C‐shaped maxillary second molar. Fourthly, a normative follow‐up schedule should involve assessments at 3, 6, 9, and 12 months, postoperatively, followed by annual visits thereafter. Owing to the patient′s personal circumstances, the in‐clinic follow‐up assessments in this study only included the 4‐month and 6‐year postoperative time points. Fifthly, genetic factors were not investigated in the present case. Future studies are expected to explore the precise impact of genetic factors on the occurrence of C‐shaped maxillary second molar.

## 4. Conclusion

This case report details the nonsurgical endodontic therapy of a rare C‐shaped MSM with five canals. The adjunctive use of passive ultrasonic irrigation can help in cleaning canal systems. Clinicians should pay attention to the C‐shaped morphological variations during the endodontic treatment of maxillary molars, even though these variations infrequently occur. The presence of extra canals should always be considered during root canal treatment, and CBCT imaging coupled with a dental operating microscope will aid in their detection.

## Author Contributions

Conceptualization: Dan Zhao. Methodology: Yiwen Wang and Beining Liao. Investigation: Beining Liao and Hongyin Ruan. Data curation: Yiwen Wang and Beining Liao. Writing—original draft: Dan Zhao. Writing—review and editing: Dan Zhao and Xinyan Ge. Visualization: Yiwen Wang and Beining Liao. Supervision: Dan Zhao. Project administration: Dan Zhao.

## Funding

No funding was received for this manuscript.

## Ethics Statement

The patient has signed the informed consent form for publication. The study has been approved by the Ethics Committee of the Stomatological Hospital of Wuhan University, with the approval number WDKQ2020B07.

## Conflicts of Interest

The authors declare no conflicts of interest.
